# *In vivo* evaluation of a biodegradable intraanastomotic membrane in a porcine model

**DOI:** 10.3389/fsurg.2026.1746520

**Published:** 2026-02-09

**Authors:** Daniel C. Freund, Dennis Wahl, Eberhard Grambow, Finn Jaekel, Julia Henne, Richard Kantelberg, Hans Kleemann, Friedrich Prall, Amelie R. Zitzmann, Brigitte Vollmar, Jochen Hampe, Karl Leo, Sebastian Hinz, Clemens Schafmayer

**Affiliations:** 1 Department of General, Visceral, Thoracic, Vascular and Transplantation Surgery, Rostock University Medical Center, Rostock, Germany; 2 Department of Cardiac, Thoracic and Vascular Surgery, University Medical Center Göttingen, Göttingen, Germany; 3 Dresden Integrated Center for Applied Physics and Photonic Materials (IAPP), Technical University of Dresden, Dresden, Germany; 4 Institute of Pathology, University Medical Center Rostock, Rostock, Germany; 5 Department of Anesthesiology, Intensive Care Medicine and Pain Therapy, University Medical Centre of Rostock, Rostock, Germany; 6 Rudolf-Zenker-Institute for Experimental Surgery, University Medical Center Rostock, Rostock, Germany; 7 Department of Medicine I, University Medical Center Dresden, Technische Universität Dresden, Dresden, Germany

**Keywords:** anastomotic healing, anastomotic leak, biodegradable, *in vivo*, PDO membrane, porcine model, small intestine

## Abstract

**Background:**

Anastomotic leakage (AL) represents one of the most serious complications in gastrointestinal surgery, with reported incidence rates of up to 26%. Despite advancements in surgical techniques, early detection of AL remains challenging, and no reliable real-time monitoring system is currently available. In this study, we investigated a resorbable polydioxanone (PDO) membrane as a potential substrate for future sensor integration, aiming to facilitate real-time monitoring of anastomotic healing.

**Methods:**

In eight German Landrace pigs, 34 ileal side-to-end stapler anastomoses were examined: GM1 (*n* = 7), GM2 (*n* = 10), and controls (*n* = 17). Membrane stability was monitored after implantation, while adhesion formation, burst pressure, and histology were assessed on postoperative day 7.

**Results:**

Both membrane geometries showed robust stability, with good anchorage of the large spokes within the anastomosis. Geometry 1 (GM1) exhibited higher burst pressure than Geometry 2 (GM2) (193 ± 43.6 vs. 155 ± 65.5 mmHg, *p* = 0.02). Compared with controls (167 ± 42.3 mmHg), neither GM1 (*p* = 0.053) nor GM2 (*p* = 0.379) differed significantly. Adhesions occurred in all groups, without significant differences. Histological evaluations showed typical granulation tissue and fibrosis, with granulocytic inflammation more common in GM1 without affecting anastomotic stability.

**Conclusion:**

This proof-of-concept study demonstrates that the PDO membrane can be safely incorporated into stapled anastomoses without compromising anastomotic healing. The membrane provides a stable, biocompatible platform suitable for future sensor integration, supporting the development of a diagnostic intraanastomotic device.

## 1 Introduction

Anastomotic leakage (AL) remains one of the most serious postoperative complications in visceral surgery, with incidence rates of up to 26%, leading to significantly increased patient morbidity and mortality (1–3). In addition, AL is associated with prolonged hospitalization and substantial economic burden (4). Although early detection of anastomotic complications is critical, it remains a major clinical challenge. On average, AL is diagnosed five to eight days postoperatively (5–7), typically based on clinical signs and nonspecific laboratory parameters, such as elevated inflammatory markers. Early diagnosis has been shown to markedly improve patient outcomes (8, 9). Currently, no reliable methods exist for early detection of AL (10, 11). The aim of this study was to develop a resorbable membrane as a potential platform for future integration of sensors, enabling real-time intraanastomotic monitoring of impaired healing, facilitating timely interventions, and potentially preventing progression to full AL. Polydioxanone (PDO) was selected for membrane fabrication due to its well-established biocompatibility and controllable hydrolytic degradation (12). Moreover, the PDO membrane demonstrated moderate stability and high elasticity, with a Young's modulus of 958 MPa, indicating adequate mechanical performance for surgical applications (13). In addition, PDO retains approximately 70% of its initial strength during the first three weeks and about 58% after four weeks and is fully resorbed after six months (14, 15). Furthermore, in our previous work we demonstrated that the PDO membrane exhibits a yield stress of approximately 25 MPa at around 20% strain, confirming its characteristic combination of stiffness and ductility (16). We hypothesized that incorporation of a PDO membrane into side-to-end stapled anastomoses would not compromise healing and could provide a stable, biocompatible scaffold for future sensor integration.

## 2 Materials and methods

### 2.1 Membrane design

**Figure 1:**
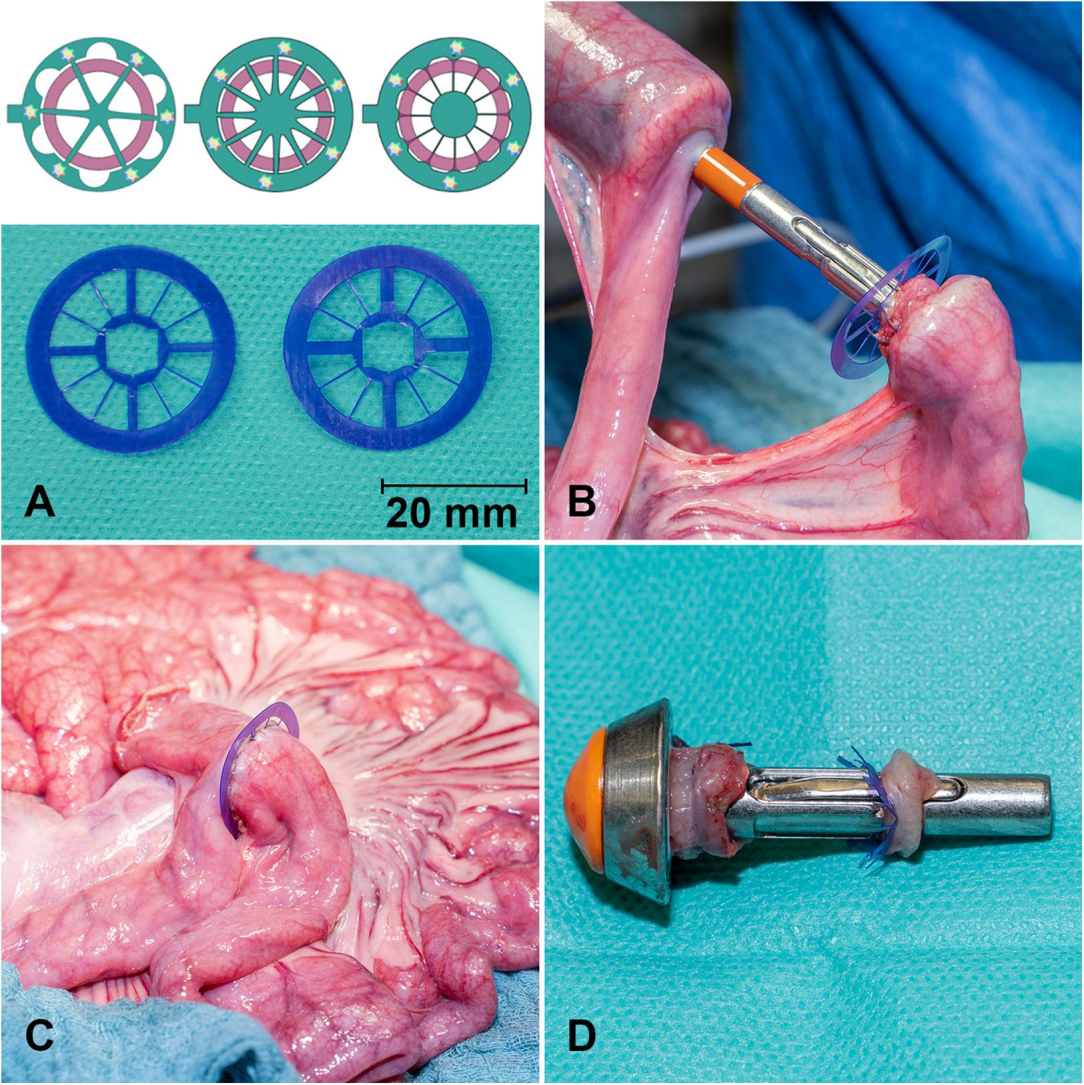
Schematic and macroscopic images of the PDO membrane with different geometries and spokes for fixation in the small bowel anastomosis. The lower photo displays GM1 (left) and GM2 (right). Each design includes four large and eight narrow spokes **(A)**. PDO membrane before **(B)** and after implanting in a small bowel **(C)** stapler anastomosis. The anvil of the stapler with bowel margins and inner ring of the PDO membrane **(D)**.

All membranes were fabricated from 150 µm thick PDO sheets (Ethicon, Inc., a Johnson & Johnson company, Somerville, NJ, USA). Following preliminary *in vitro* testing of multiple design concepts (Figure 1A, top), two designs were selected for their favorable tissue disruption ratio, structural stability, and compatibility with the stapling device. These geometries were subsequently evaluated in the present *in vivo* study (Figure 1A, bottom).

### 2.2 Animal model

The experimental cohort consisted of eight male German Landrace pigs, aged 12–16 weeks and weighing 31.6–41.0 kg (mean ± SD: 36.0 ± 3.2 kg). Another animal died prematurely due to surgical error and was excluded from the study. Post-mortem examination identified small bowel torsion proximally to the anastomotic sites. The anastomoses showed no abnormalities. Animals were housed under standardized conditions at the Central Animal Care Facility of Rostock University Medical Center and acclimatized for seven days prior to the procedure. Water was provided *ad libitum* until the day before the procedure. Premedication and anesthesia were administered according to the standard protocols of the Institute for Experimental Surgery at Rostock University Medical Center. All animal experiments were approved by the German local authority: Landesamt für Landwirtschaft, Lebensmittelsicherheit und Fischerei Mecklenburg-Vorpommern (approval no. 7221.3-1-050/19), under the German animal protection law and the NIH Guide for the Care and Use of Laboratory Animals (Institute of Laboratory Animal Resources, National Research Council) (17).

### 2.3 Sedation and anesthesia

Premedication was administered intramuscularly with 8 mg/kg azaperone (Stresnil®, Elanco, Cuxhaven, Germany), 20 mg/kg ketamine (10% Ketamin, Medistar Arzneimittelbetrieb GmbH, Ascheberg, Germany) and 0.2 mg/kg midazolam (Dormicum, Hoffmann La Roche AG, Grenzach-Wyhlen, Germany). Animals were equipped with a pulse oximeter (Nellcor® PM10N, Medtronic, Watford, UK) placed on the tail and two peripheral venous cannulas (20G, B. Braun Melsungen AG, Melsungen, Germany) in the earlobe veins. Anesthesia was induced with 200 µg fentanyl (Fentadon® 50 µg/mL, Eurovet Animal Health BV, Bladel, Netherlands), 100 mg propofol (Propofol 2%, MCT Fresenius, Bad Homburg, Germany) and 4 mg pancuronium (Pancuronium Inresa 4 mg/2 mL, Inresa Arzneimittel GmbH, Freiburg, Germany). Maintenance anesthesia was provided via total intravenous anesthesia (TIVA) with fentanyl (5–10 µg kg^−1^ h^−1^), propofol (4–8 mg kg^−1^ h^−1^), and midazolam (0.1 mg kg^−1^ h^−1^) (18). Endotracheal intubation was performed using a 7 mm inner-diameter tube, followed by volume-controlled ventilation with a Dräger Primus® ventilator, while continuously monitoring oxygen saturation, heart rate, respiratory minute volume, and end-tidal CO_2_. Following induction, animals were placed in the supine position on the operating table and secured at all extremities.

### 2.4 Surgical model

After sterile preparation using Braunol® (B. Braun Melsungen AG, Melsungen, Germany), a midline laparotomy was performed with a careful right-sided incision around the urethra. Anastomoses were created at intervals of approximately 50 cm oral to the ileocecal valve. Side-to-end stapler anastomoses were constructed in the small intestine, allowing multiple anastomoses per animal to reduce the number of animals required for the study. After an initial mesenteric incision between the marginal arteries, the bowel was transected using monopolar cautery (ICC 300, Erbe Elektromedizin, Tübingen, Germany). The stapler anvil was inserted into the oral end of the bowel and secured with a preplaced purse-string suture (Vicryl 3.0, Ethicon®, Inc., a Johnson & Johnson company, Somerville, NJ, USA). The stapler (21 mm Ethicon^™^ Circular Stapler, Ethicon®, Inc., a Johnson & Johnson company, Somerville, NJ, USA) was then introduced into the aboral end, and the intestine was pierced with the trocar opposite the mesenteric side. The PDO membrane was positioned on the stapler trocar, the stapler components were connected (Figure 1B), and the intestinal ends were approximated by gradually closing the stapler. The stapler was then fired, excising the inner membrane section to ensure anastomotic patency and prevent luminal obstruction (Figure 1D). Following stapler removal, the blind end was resected, leaving approximately 1 cm of bowel to prevent ischemia around the anastomosis, and closed with a running suture (PDS 4-0, Ethicon®, Inc., a Johnson & Johnson company, Somerville, NJ, USA (Figure 1C). In total, 34 ileal side-to-end anastomoses were examined, subdivided into three groups: GM1 (*n* = 7), GM2 (*n* = 10), and control (*n* = 17). Membranes were inspected for spoke displacement, and the intestines were repositioned. The abdominal wall was closed with a fascial suture (PDS sling 1.0, Ethicon®, Inc., a Johnson & Johnson company, Somerville, NJ, USA) and a skin staple closure (Disposable Skin Stapler F35w, ADVAN, China), followed by application of silver-aluminum spray to the wound. Depending on intraabdominal conditions, three to five anastomoses were performed per animal. Postoperatively, animals received water *ad libitum* and a standardized diet (2 × 300 g MPig-H, ssniff®, Soest, Germany). Oral analgesia was provided daily with 2 g metamizole (Novaminsulfon 500 mg/mL, Winthrop Arzneimittel GmbH, Frankfurt, Germany). Wounds were treated daily with iodine solution. Animal well-being was monitored using a standardized distress score (see Supplementary Material).

### 2.5 Relaparotomy, macroscopic evaluation and burst pressure measurement

**Figure 2:**
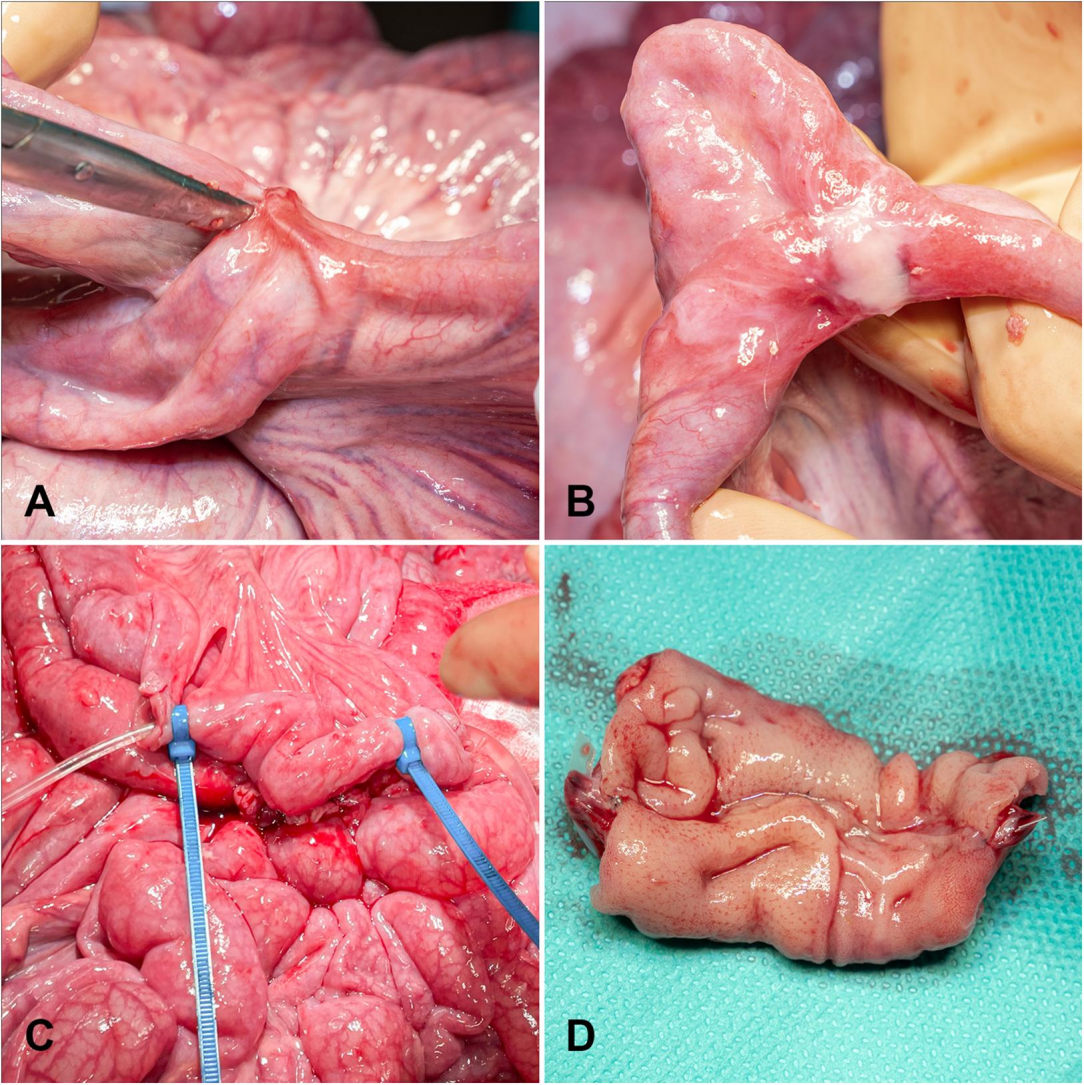
Postoperative macroscopic analysis of small bowel anastomoses on day seven. The scissors point to an adhesion (2+) **(A)**. Adhesion and fibrin deposition (1+) **(B)**. For burst pressure analysis, catheters for saline infusion (left tube) and pressure analysis (right tube) were inserted near the anastomosis and secured using cable ties **(C)** Resected anastomosis for macroscopical study after midline incision **(D)**.

Relaparotomy was scheduled on postoperative day seven. One animal underwent relaparotomy on postoperative day five due to an elevated distress score (apathy and immobility), in accordance with the predefined study criteria. No pathological findings were detected intraoperatively, and the anastomoses remained included in the analysis. One animal underwent relaparotomy on the tenth day. The abdominal cavity was inspected for signs of complications such as peritonitis, inflammation, or bowel obstruction. The anastomoses were then identified and evaluated for macroscopic integrity and adhesion formation. Adhesions were graded according to the van der Ham score (19) as follows: 0 = no adhesions; 1+ = minimal adhesions, primarily between the anastomosis and the omentum; 2+ = moderate adhesions, involving the omentum, anastomotic site, and adjacent small bowel loops; and 3+ = severe and extensive adhesions, including abscess formation (Figures 2A,B). For assessment of anastomotic burst pressure, the bowel was incised 5 cm proximal and distal to the anastomosis. A catheter was inserted for isotonic saline infusion and another for pressure measurement. Both ends were securely closed, and the catheters were sealed with cable ties (Figure 2C) (20). The bowel segment was then continuously filled until rupture occurred, and the peak intraluminal pressure was recorded (21). The rupture site was documented as occurring either at the anastomosis or at a distant bowel segment. Finally, while under general anesthesia, animals were euthanized with 45 mg·kg^−^¹ pentobarbital (Release® 300 mg/mL, Wirtschaftsgenossenschaft Deutscher Tierärzte eG., Garbsen, Germany).

### 2.6 Histological analysis

Intestinal segments containing the anastomoses were excised, opened along the antimesenteric border (Figure 2D), and fixed in a stretched position in 10% buffered formalin for 24 h (22). After careful removal of the metallic stapler clips, two longitudinal tissue sections, one from the mesenteric and one from the antimesenteric side, were obtained from each specimen (approximately 25 mm in length and 4 mm in thickness), with the anastomotic site positioned centrally (23). The samples were embedded in paraffin and sectioned into 4 µm slices using a microtome (Leica RM 2145). All sections were stained with hematoxylin and eosin (H&E) and examined by a surgical pathologist blinded to the group (with or without membrane).

### 2.7 Statistical analysis

All statistical analyses were performed using IBM SPSS Statistics for Windows, Version 29.0.2.0 (IBM Corp., Armonk, NY, USA). The Mann–Whitney *U* test was applied to assess differences in burst pressure between groups. The Fisher–Freeman–Halton test was used to evaluate the significance of all other data, and the Jonckheere–Terpstra test was applied to analyze the van der Ham adhesion scores. Continuous variables are presented as medians. A *p*-value < 0.05 was considered statistically significant.

## 3 Results

### 3.1 Membrane design and stability

**Figure 3:**
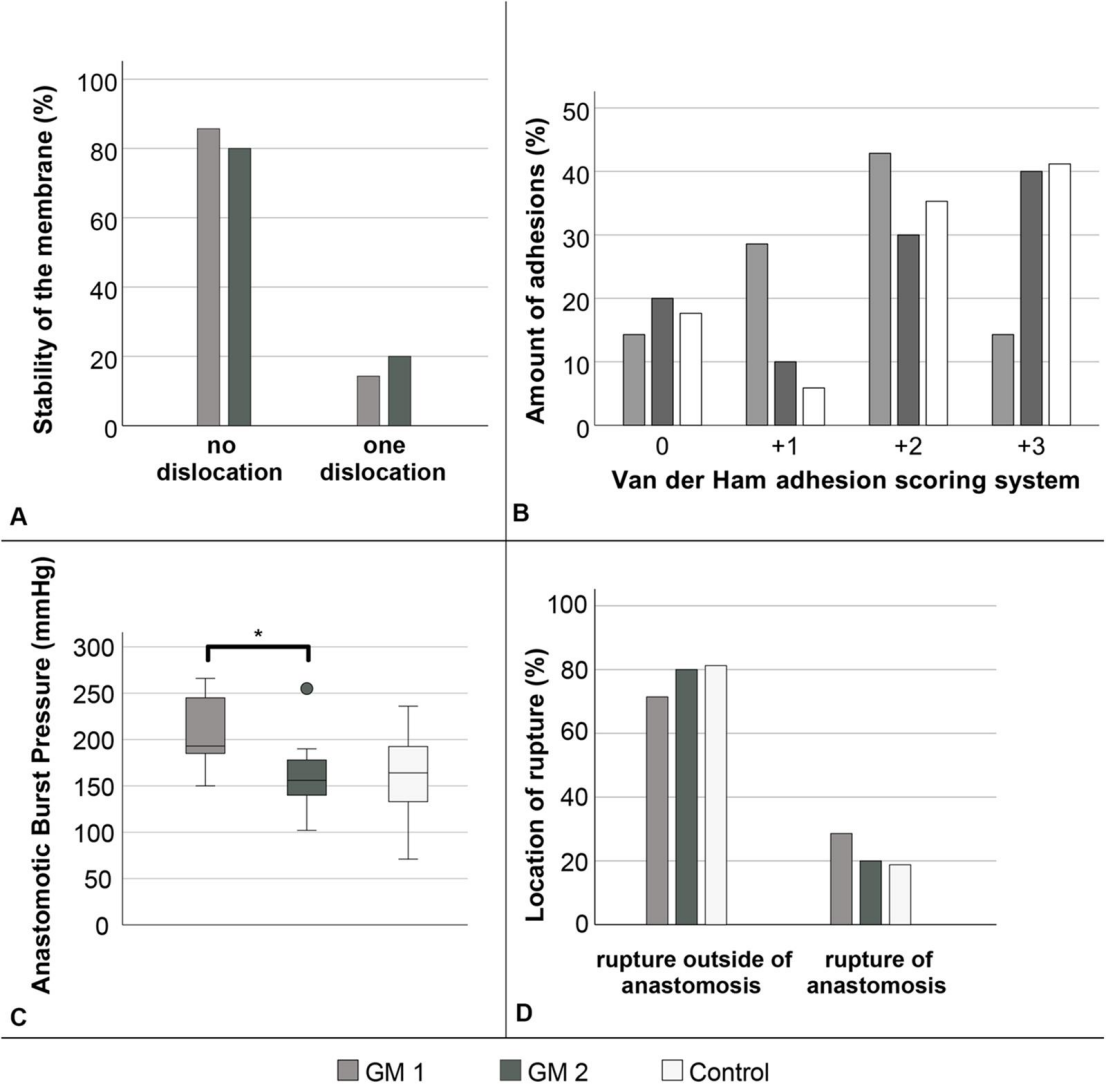
Quantitative analysis of PDO membrane stability immediately after implantation in a small bowel stapler anastomosis. Stability was assessed by dislocation large spokes of the membrane. No significant differences between the groups (*p* = 0.640; Fisher–Freeman–Halton test) **(A)**. Analysis of anastomotic stability. Quantitative assessment of van der Ham adhesion score based on adhesion formation around the anastomosis showed no significant difference (*p* = 0.358; Jonckheere–Terpstra test) **(B)**. Stability was further studied by means of burst pressure; the difference between GM1 and GM2 was significant (*p* = 0.02; Mann–Whitney *U* test), while no significant differences were observed compared with the control group (GM1 vs. Control: *p* = 0.053; GM2 vs. Control: *p* = 0.379) **(C)**. The location of rupture during burst pressure assessment showed no significant difference (*p* > 0.05; Fisher–Freeman–Halton test) **(D)**. GM1: *n* = 7, GM2: *n* = 10, Control: *n* = 17.

After initial design optimization, two membrane prototypes were evaluated *in vivo*. Both designs comprised an outer ring (28.88 mm outer diameter, 21.38 mm inner diameter) connected to an inner ring (10 mm outer diameter) by eight narrow and four wide spokes (Figure 1A). GM1 featured 1.5 mm-wide spokes, while GM2 incorporated 2.0 mm-wide spokes; both designs included 0.6 mm narrow spokes. A central circular cutout (6.4 mm) accommodated the trocar of a 21 mm circular stapler, with additional peripheral cuts aligned to the trocar's widest points to prevent rotation or dislocation during implantation. The spoke configuration was designed to maximize surface area for future sensor integration while maintaining stable anchoring and minimizing the amount of material embedded within the intestinal wall to reduce interference with anastomotic healing. After stapled implantation, membranes were examined for dislocation or structural damage (Figure 3A). Outer ring damage was observed in one GM1 anastomosis. Dislocation of large spokes was infrequent (GM1: 14.3%; GM2: 20.0%; *p* = 0.640; Figure 3A). Dislocation of small spokes occurred in both geometries: in GM1, 43.9% of anastomoses showed all small spokes intact, none exhibited single spoke dislocation, and 57.1% showed dislocation of two spokes; in GM2, 40.0% had all spokes intact, 30.0% exhibited dislocation of one spoke, and 30.0% showed dislocation of two spokes. There was no statistically significant difference in the rate or extent of small spoke dislocation between groups (*p* = 0.358).

### 3.2 Macroscopic examination

No AL or stenosis were observed in any group. All anastomoses were patent and free of stenosis. Adhesions were present in all groups, with variable severity (Figures 2A,B, 3B). In GM1, 14.3% of anastomoses showed no adhesions, 28.6% had mild adhesions (+1), 42.9% moderate (+2), and 14.3% severe (+3). In GM2, 20% were adhesion-free, 10% mild, 30% moderate, and 40% severe. In the control group, 17.6% showed no adhesion, 5.9% mild, 35.3% moderate, and 41.2% severe. Mild adhesions were more frequent in GM1, whereas severe adhesions predominated in GM2 and controls. Overall, differences in adhesion distribution among groups were not statistically significant (*p* = 0.358).

### 3.3 Anastomotic burst pressure

Anastomotic burst pressure was highest in GM1 (193 ± 43.6 mmHg) and lowest in GM2 (155 ± 65.5 mmHg), compared with 167 ± 42.3 mmHg in the control group (Figure 3C). The difference between GM1 and GM2 reached statistical significance (*p* = 0.02), whereas comparisons of GM1 or GM2 with controls were not significant (*p* = 0.053 and *p* = 0.379, respectively). In most cases, intestinal rupture occurred outside the anastomosis (71.4% in GM1, 80% in GM2, and 82.4% in controls; *p* = 0.861; Figure 3D), with no notable differences between groups.

### 3.4 Histological examination

**Figure 4:**
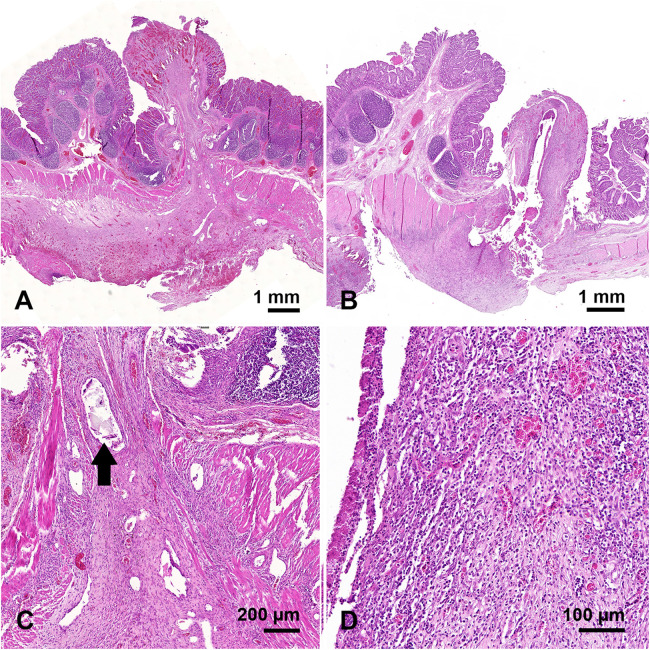
Microscopic images of two anastomoses, both from group GM2: **(A)** panoramic view of an anastomosis without significant granulocytic inflammation; **(C)** at higher magnification residual PDO substrate is seen (arrow) which is surrounded by a fibrosing reaction. **(B)** Panoramic view of an anastomosis with substantial granulocytic inflammation (scored GIS) around residual PDO substrate, which at higher magnification **(D)** is fully appreciated.

78 specimens were submitted to histological examination and the anastomoses were visualized in 77 of these. Granulation tissue and fibroblast-rich fibrosis were found in all anastomoses, frequently in zonal arrangements and often surrounding residual surgical material (Figures 4A,C). However, differences between anastomoses were observed as follows and scored in a systematic slide review as present or absent: granulocytic inflammation adjacent to surgical material (GIS) (Figures 4B,D); abscess formation independent of residual surgical material (IAF); surgical induced mucosal hernias without abscess formation (HAS), abscess formation in surgically induced mucosal hernias (HWA); purulent exudate on the peritoneum (PEP). The slide review was done blinded to anastomosis types and the type of membrane (GM1 vs. GM2). The results of the two slices per anastomosis were combined into one result, basing the final score on the more severe histological finding. GIS was found more frequently in the groups of the membranes, especially in group GM1 (GM1: 42.9%, GM2: 30.0% Control: 11.8%; *p* = 0.221). PEP also occurred most frequently in GM1 and was least frequent in the control group (GM1: 42.9%, GM2: 10.0%, Control: 5.9%; *p* = 0.076). In contrast, HWA was found exclusively in the control group (Table 1). However, the differences between the three groups were not statistically significant.

**Table 1:** Results of the histological examination: granulocytic inflammation adjacent to surgical material (GIS); abscess formation independent of residual surgical material (IAF); surgical induced mucosal hernias without abscess formation (HAS), abscess formation in surgically induced mucosal hernias (HWA); purulent exudate on the peritoneum (PEP). The difference between the groups does not reach statistical significance (all *p*-values > 0.05; Fisher-Freeman-Halton test).

Histological findings	GM 1 Group	GM 2 Group	Control Group	*p*-value
	in (%)	in (%)	in (%)	
GIS	42.9	30.0	11.8	0.221
IAF	14.3	10.0	5.9	0.773
HAS	0	20.0	5.9	0.415
HWA	0	0	11.8	0.697
PEP	42.9	10.0	5.9	0.076

## 4 Discussion

### 4.1 Principal findings

This study suggests that integration of a PDO membrane into stapled anastomoses does not impair anastomotic healing, supporting its potential as an intraanastomotic carrier membrane for sensor devices. GM1 exhibited slightly higher macroscopic healing performance than GM2, although this difference reached statistical significance only in burst pressure measurements. No group performed worse than comparable data reported in the literature (20). All other macroscopic parameters were similar across the groups. Moderate to severe adhesions were observed in all animals, which may partly reflect the relatively high number of anastomoses created per animal in this 3R-adapted study design. As no membrane was implanted in the control group, the severe adhesions most likely represent the typical postoperative response to stapler-based small bowel anastomoses rather than an effect attributable to the PDO membrane. The comparable adhesion severity in GM2 therefore suggests that the membrane did not exacerbate adhesion formation. Although these adhesions did not impair anastomotic integrity within the seven-day observation period, postoperative adhesions remain a major challenge in abdominal surgery and may still carry clinical relevance (24, 25). The long-term evolution and implications of these findings warrant further investigation in extended follow-up models. Histological analysis indicated a higher frequency of granulocytic inflammation and purulent exudate in GM1 compared with GM2 (Table 1), suggesting a modest local tissue response. However, these differences were not statistically significant and likely reflect a physiological reaction to resorbable material, without impacting anastomotic stability. As shown previously, the initial inflammation observed around the PDO membrane represents a typical foreign-body reaction. Furthermore, it has been demonstrated that this response progressively decreases over time and can be classified as very slight after 3–6 months (26, 27). PDO has a long history of clinical use as a suture and implant material, with well-established biocompatibility and predictable *in vivo* degradation kinetics (12). This study confirms the mechanical stability of the membrane and its compatibility with standard surgical techniques. The membrane could be reliably introduced through the circular stapler without technical difficulty, and the stapler functioned normally with the membrane in place. Large spokes remained stable, supporting potential future circumferential sensing. The outer ring was largely intact, with a single break in GM2 resulting from moisture-related accelerated PDO (12) degradation due to non-airtight storage, which could be prevented with improved industrial packaging. Dislocation of small spokes occurred frequently but was not considered critical, as they primarily support implantation and are not intended for sensor integration.

### 4.2 Clinical relevance and translational outlook

AL is influenced by multiple factors, some modifiable intraoperatively (e.g., blood loss, fecal contamination, operative time), and others patient-specific (e.g., chronic kidney disease, diabetes, hypertension, smoking), which are prognostic for AL (28–32). These factors often affect microvascular perfusion and wound healing, meaning not all AL can be prevented. Non-technically induced insufficiencies typically develop gradually, offering an opportunity for early detection prior to perforation or peritonitis. Currently, no clinical device provides real time, direct monitoring of anastomotic healing. Radiologic methods, such as CT imaging, have limited sensitivity, as shown by Doeksen et al. (33, 34), and involve radiation exposure that should be avoided without clinical indication (34, 35). Endoscopic examination provides sufficient sensitivity and specificity but is invasive. Recent studies have explored bioresorbable sensors, including impedance-based systems (36), magnesium electrodes (37), and wireless oxygen or pH sensors (38). However, these approaches generally provide only single-point measurements or require external positioning, limiting circumferential coverage. In contrast, the PDO membrane provides a mechanically stable, circumferential platform for potential site-specific sensing along the entire staple line. At the same time, several studies have already shown that impedance measurements can be used to recognize anastomotic insufficiencies at an early stage (39, 40). Previous *ex vivo* work demonstrated the feasibility of integrating resorbable electronic elements directly onto PDO membranes using screen-printable zinc and silver inks, confirming mechanical stability, biocompatibility, and controlled degradation (16). Future studies will focus on incorporating sensors (impedance-, oxygen-, or lactate-based) (41–43) onto the membrane surface to enable continuous, localized monitoring of anastomotic healing. The circumferential design may allow early detection of impaired healing, supporting timely intervention. From a translational perspective, the use of clinically established PDO and standard manufacturing techniques such as extrusion or thermoforming enables scalable, cost-efficient production of sensor-integrated membranes compatible with existing medical-grade polymers once sensor functionality and long-term safety have been validated. This study demonstrates that a bioresorbable PDO membrane can be safely integrated into stapler anastomoses in a porcine model without compromising anastomotic healing. The membrane provides a mechanically stable, biocompatible platform suitable for future development of smart anastomotic devices.

### 4.3 Limitations

The obtained results are part of a proof-of-concept study; therefore, the sample size was not calculated by a power analysis. To finally evaluate the influence on long-term anastomotic healing, a power analysis-based case-control study should be performed including extended clinical and histological follow-up. In our study, granulocytic inflammation adjacent to residual PDO material occurred more frequently in the membrane groups, particularly in GM1, although without compromising anastomotic stability (Table 1). This response is consistent with the early inflammatory phase typically associated with resorbable polymers but highlights the need to further optimize membrane geometry and material load to minimize local irritation. Mechanical factors such as spoke dislocation may also contribute to localized tissue stress and should be addressed through improved design refinement. This may also help to reduce adhesion formation. Consequently, the translational potential to human application remains preliminary. Future studies should address sensor material integration and prolonged observation periods.

## 5 Conclusion

This proof-of-concept study demonstrates the technical feasibility of integrating a resorbable PDO membrane into stapled small bowel anastomoses without compromising anastomotic integrity, thereby highlighting its potential for future sensor integration. The results are encouraging, but further studies with larger cohorts and extended follow-up are required to confirm safety beyond a reasonable doubt.

## Data Availability

The raw data supporting the conclusions of this article will be made available by the authors, without undue reservation.
