# Supplementary material for: In vivo evaluation of a biodegradable intraanastomotic membrane in a porcine model
Source: Front Surg. 2026 Feb 9;13:1746520. doi: 10.3389/fsurg.2026.1746520 (PMC12926394; doi:10.3389/fsurg.2026.1746520)
Supplement: Supplementary file 1 [file Table1.docx]

Supplementary Material

# Supplementary Figures and Tables

## Supplementary Table 1 - Score Sheet

| I Observation | Score |
| --- | --- |
| Hemodynamic instability with persistent need for vasopressors | 1 |
| Hypoxia with SpO_2_ saturation below 50% lasting more than 5 minutes | 1 |
| Persistent uncontrollable bleeding | 3 |
| II General Conditions | Score* |
| Scaly skin; cloudy eyes | 1* |
| Clogged body openings; abnormal posture; dehydration | 1* |
| Seizures, paralysis, abnormal respiratory sounds, hypothermia | 2* |
| III Spontaneous Behavior | Score* |
| Isolation | 1* |
| Apathy (almost no voluntary movement observable) | 2* |
| Signs of pain | 3* |
| Automutilation | 3* |
| IV Procedure-Specific Criteria | Score* |
| Open wounds | 1* |
| **Total Theoretical Score** | **0-19** |
| **Supplementary Table 1:** Clinical observation and procedural scoring system for welfare assessment in a porcine model. Parameters include hemodynamic stability, oxygenation, bleeding control, general condition, and spontaneous behavior. Scores were used to determine severity classification and to trigger predefined interventions or humane endpoints in accordance with EU Directive 2010/63/EU, FELASA recommendations, and institutional animal welfare regulations. | |

## Supplementary Table 2 - Interventions and Endpoints

| \| **Individual Score** \| **Total Score** \| **Burden** \| **Intervention** \| \| --- \| --- \| --- \| --- \| \| * \| - \| - \| The experiment is not initiated. \| \| 1 (IV) \| 1 \| mild \| Wound revision under general anesthesia. \| \| 3 (III) \| 3-9 \| severe \| The experiment is terminated by intravenous administration of pentobarbital, **or** the animal is anesthetized and re-laparotomized prior to euthanasia. \| \| 1-2 (III) \| 1-3 \| moderate \| Clinical reevaluation of spontaneous behavior after 60 min. If the score remains unchanged, anesthesia is induced and a re-laparotomy is performed, followed by euthanasia. \| \| 2 \| 2-4 \| severe \| The animal is anesthetized and re-laparotomized prior to euthanasia. \| \| 1 \| 1-2 \| mild \| Clinical reevaluation after 4 hours. If the score remains unchanged, anesthesia and re-laparotomy are performed and euthanasia is carried out in the presence of anastomotic leakage or bowel gangrene. \| \| 1 \| 1 \| mild \| Optimization of fluid therapy; surgical site revision if indicated. \| \| 3 (I) \| 3-5 \| severe \| The experiment is terminated by intravenous administration of pentobarbital. \| |
| --- | --- | --- | --- | --- | --- | --- | --- | --- | --- | --- | --- | --- | --- | --- | --- | --- | --- | --- | --- | --- | --- | --- | --- | --- | --- | --- | --- | --- | --- | --- | --- | --- | --- | --- | --- | --- |

**Supplementary Table 2**: Defined interventions and endpoints based on individual and cumulative clinical scores. Interventions ranged from supportive management to re-laparotomy and euthanasia using pentobarbital injection where indicated. Animals exceeding threshold scores or failing to improve on reevaluation were anesthetized and humanely euthanized prior to awakening.
